# Supplementary material for: Diagnostic Utility of Metalloproteinases from Collagenase Group (MMP-1, MMP-8 and MMP-13) in Biochemical Diagnosis of Ovarian Carcinoma
Source: Cancers (Basel). 2024 Nov 26;16(23):3969. doi: 10.3390/cancers16233969 (PMC11640008; doi:10.3390/cancers16233969)
Supplement: Supplementary file 1 [file cancers-16-03969-s001.zip › cancers-3312500-supplementary.pdf]

Table S1. The Spearman's rank correlation test for MMP-1. MMP-8. MMP-13 and HE4 and CA125 in tested groups.

	Tested Correlations														
	MMP-1	MMP-1	MMP-1	MMP-1	MMP-1	MMP-8	MMP-8	MMP-8	MMP-8	MMP-13	MMP-13	MMP-13	CA125	CA125	HE4
	vs														
	MMP-8	MMP-13	CA125	HE4	ROMA	MMP-13	CA125	HE4	ROMA	CA125	HE4	ROMA	HE4	ROMA	ROMA
**Ovarian Carcinoma**															
**r**	0.168	0.074	0.111	-0.027	0.049	-0.044	0.045	-0.134	-0.002	-0.002	0.093	0.021	0.374	0.739	0.849
**P**	0.066035	0.420192	0.22348	0.76814	0.593446	0.632238	0.624687	0.144024	0.981861	0.98018	0.30881	0.814484	0.000025	<0.000001	<0.000001
**Begin Ovarian Lesion**															
**r**	-0.021	-0.013	0.028	0.126	0.041	0.082	-0.118	0.145	-0.003	-0.212	0.220	-0.019	0.137	0.498	0.631
**P**	0.856808	0.91168	0.81563	0.29584	0.73127	0.498826	0.32998	0.230103	0.974656	0.077346	0.067089	0.873998	0.25625	0.000011	<0.000001
**Healthy subjects**															
**r**	-0.093	-0.113	0.07	0.066	0.048	0.053	0.023	-0.081	0.008	-0.068	-0.254	-0.235	0.160	0.736	0.727
**P**	0.520088	0.430578	0.62568	0.64613	0.73623	0.712057	0.87044	0.575673	0.952485	0.636794	0.074039	0.099793	0.26546	<0.000001	<0.000001

Red color indicates statistically significant correlations of tested parameters.
